# The Sp1 transcription factor is essential for the expression of gliostatin/thymidine phosphorylase in rheumatoid fibroblast-like synoviocytes

**DOI:** 10.1186/ar3811

**Published:** 2012-04-25

**Authors:** Kenji Ikuta, Yuko Waguri-Nagaya, Kae Kikuchi, Takaya Yamagami, Masahiro Nozaki, Mineyoshi Aoyama, Kiyofumi Asai, Takanobu Otsuka

**Affiliations:** 1 Department of Orthopedic Surgery, Nagoya City University Graduate School of Medical Sciences, 1 Kawasumi, Mizuho-cho, MuzuhoMizuho-ku, Nagoya, 467-8601, Japan; 2 Department of Molecular Neurobiology, Nagoya City University Graduate School of Medical Sciences, Mizuho-Ku, 1 Kawasumi, Mizuho-cho, MuzuhoMizuho-ku, Nagoya, 467-8601, Japan

## Abstract

**Introduction:**

Gliostatin/thymidine phosphorylase (GLS/TP) has angiogenic and arthritogenic activities, and aberrant GLS production has been observed in the active synovial membranes of rheumatoid arthritis (RA) patients. The human GLS gene promoter contains at least seven consensus binding sites for the DNA binding protein Sp1. Here we examined whether Sp1 is necessary for GLS production in RA. We also studied the effects of the Sp1 inhibitor mithramycin on GLS production in RA fibroblast-like synoviocytes (FLSs).

**Methods:**

FLSs from RA patients were treated with specific inhibitors. The gene and protein expression of GLS were studied using the quantitative reverse-transcription polymerase chain reaction (qRT-PCR) and an enzyme immunoassay. Intracellular signalling pathway activation was determined by western blotting analysis, a luciferase assay, a chromatin immunoprecipitation (ChIP) assay and a small interfering RNA (siRNA) transfection.

**Results:**

The luciferase and ChIP assays showed that Sp1 binding sites in the GLS promoter were essential for GLS messenger RNA (mRNA) expression. GLS production was suppressed in FLSs by siRNA against Sp1 transfection. Mithramycin decreased GLS promoter activity, mRNA and protein expression in FLSs. Tumour necrosis factor-α (TNF-α) significantly increased GLS expression in RA FLSs; this effect was reduced by pre-treatment with cycloheximide and mithramycin.

**Conclusions:**

Pretreatment of mithramycin and Sp1 silencing resulted in a significant suppression of GLS production in TNF-α-stimulated FLSs compared to controls. GLS gene expression enhanced by TNF-α was partly mediated through Sp1. As physiological concentrations of mithramycin can regulate GLS production in RA, mithramycin is a promising candidate for anti-rheumatic therapy.

## Introduction

Many components of the immune system contribute to the development and progression of rheumatoid arthritis (RA), and angiogenesis is considered a critical step in its initiation and progression. Levels of inflammatory cytokines such as tumor necrosis factor-alpha (TNF-α) and interleukin-1-beta (IL-1β) and IL-6 [1-3] are increased in arthritic joints, whereas high levels of angiogenic factors such as vascular endothelial growth factor (VEGF) [4,5] and gliostatin (GLS) [6,7] have been reported in synovial fluid and sera from patients with RA. For this reason, the past decade has seen the development of biological treatments for RA, such as TNF-α inhibitors.

GLS is thought to be similar to thymidine phosphorylase (TP) and platelet-derived endothelial cell growth factor (PD-ECGF) [8] and induces angiogenesis through the proliferation and chemotactic migration of endothelial cells [9-11]. It also inhibits the growth of glial cells [12], promotes glial cell differentiation, and has neurotrophic effects on cortical neurons [13]. GLS production by cultured RA fibroblast-like synoviocytes (FLSs) was shown to be enhanced by TNF-α [14], IL-1β [15], and GLS itself [16]. GLS was also found to induce VEGF expression in FLSs [14], and we reported that direct injection of GLS into rabbit knees led to pronounced RA-like synovitis [17]. Inhibition of GLS is therefore regarded as an important approach in reducing damage to RA tissues.

Analysis of the TP promoter region revealed several potential zinc finger-type transcription factor Sp1-binding sites, including those in numerous housekeeping and inducible genes [18], in the interferon-stimulated response element (ISRE), and in the gamma-activated sequence [19]. In the present study, we used a luciferase assay and a small interfering RNA (siRNA) against Sp1 to show that the Sp1 box is essential for GLS production. We also used the inhibitor mithramycin to demonstrate the effect of Sp1-binding inhibition on GLS expression. Mithramycin is an aureolic acid anti-neoplastic antibiotic used for treating cancer-related hypercalcemia, leukemia [20], and testicular cancer [21] and prevents Sp1 from binding to its cognate site in DNA by modifying CG sequences [22]. Here, we show that mithramycin potently suppresses GLS induction through the transduction of Sp1 in RA FLSs.

## Materials and methods

### Preparation of human fibroblast-like synoviocytes

This study was approved by the ethics committee of Nagoya City University (Nagoya City, Japan). Before participation, informed consent was obtained from all subjects in accordance with the Declaration of Helsinki. FLSs were cultured from the synovial tissues of 10 patients who had RA, who were undergoing total knee arthroplasty, and who met the American Rheumatism Association 1987 revised criteria for the classification of RA [23], as previously described [24-26]. The clinical characteristics of these patients are shown in Table 1. FLSs were maintained in Dulbecco's modified Eagle's medium supplemented with penicillin (100 units per mL), streptomycin (100 μg/mL), and 10% fetal bovine serum at 37°C in a 5% CO_2 _atmosphere. The cultures were found to be completely free of lymphoid and monocytic cells. Cells were allowed to adhere overnight, and then non-adherent cells were removed and adherent FLSs were split at a 1:3 ratio when they reached 70% to 80% confluency. FLSs were used between passages 3 and 9, during which time they were a homogeneous population of cells.

**Table 1:** Clinical characteristics of patients

Characteristic	n = 10
Gender, female/male	7/3
Age in years, range (mean)	48-74 (65.0)
Disease duration in years, range (mean)	1-18 (10.6)
CRP in mg/dL, range (mean)	0.05-3.34 (0.66)
ESR in mm/hour, range (mean)	1-83 (20.8)
Rheumatoid factor, positive/negative	9/1
Anti-CCP antibody in U/mL, range (mean)	0.6-100 (37.8)
MMP-3 in ng/mL, range (mean)	38.9-525.7 (222)
Steinbrocker stage, I/II/III/IV	0/5/3/2
Patients using DMARDs and biologics	
Methotrexate	7
Infliximab	1
Etanercept	2
FK506	2
Patients using oral steroids	5

Anti-CCP, anti-cyclic citrullinated peptide; CRP, C-reactive protein; DMARD, disease-modifying anti-rheumatic drug; ESR, erythrocyte sedimentation rate; MMP-3, matrix metalloproteinase-3.

### Luciferase assay

**Figure 1:**
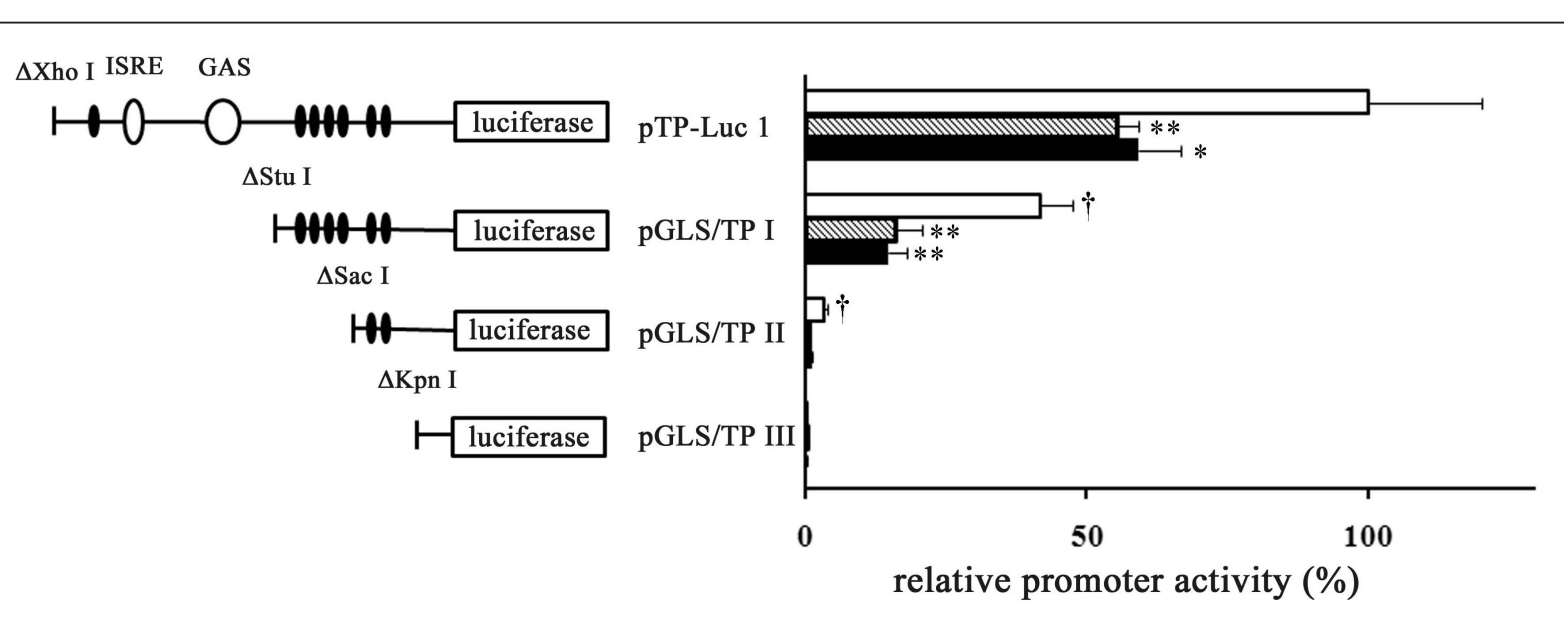
**Effects of mithramycin on gliostatin/thymidine phosphorylase (GLS/TP) promoter activity based on deletion constructs**. Closed ovals indicate Sp1-binding sites. Open ovals indicate interferon-stimulated response element (ISRE) and gamma-activated sequence (GAS) sequences. Fibroblast-like synoviocytes were transiently transfected with deletion constructs and then incubated with mithramycin (100 nM, shaded column, or 300 nM, closed column) for 24 hours. Control cells were incubated without mithramycin (open column). Data were normalized by measuring the luminescent reaction of the internal control. Results are presented as mean ± standard error of the mean of five determinations. Statistical significance was calculated by using the Mann-Whitney *U *test: compared with samples without mithramycin *P < 0.05, **P < 0.01; compared with samples with pTP-Luc1 without mithramycin ^†^*P *< 0.01.

The 1,243-base pair (bp) GLS/TP promoter fragment was a kind gift from Mayumi Ono (Department of Pharmaceutical Oncology, Kyushu University, Fukuoka, Japan) [19]. The fragment was cloned into luciferase gene Basic Vector 2 (NIPPON GENE CO., LTD, Tokyo, Japan) and digested with *Xho*I and *Hind*III. This reporter construct was designated pTP-Luc1. To prepare other deletion constructs, pTP-Luc1 was digested with different restriction enzymes, including *Xho*I, *Stu*I, *Sac*I, and *Kpn*I. Both 5' overhangs and 3' overhangs of the digested product were blunted by T4 DNA polymerase (Takara Bio Inc., Otsu, Japan) and self-ligated by T4 DNA ligase (Takara Bio Inc.) to produce pGLS/TP I, pGLS/TP II, and pGLS/TP III (Figure 1).

FLSs were seeded into a 48-well culture plate (5 × 10^3 ^cells per well) and incubated at 37°C in a humidified atmosphere of 5% CO_2_/air for 1 week. They were then co-transfected with luciferase reporter vector (400 ng/well) and pRL-SV40 internal control construct (10 ng/well) (Promega Corporation, Madison, WI, USA) by using Lipofectamine™ LTX (0.5 μL/well) and Plus™ Reagent (0.5 μL/well) (Invitrogen Corporation, Life Technologies Corporation, Carlsbad, CA, USA). The medium was replaced after 12 hours, and FLSs were treated with 100 or 300 nM mithramycin (Sigma-Aldrich, St. Louis, MO, USA) for 24 hours. Cells were harvested by using passive lysis buffer (65 μL/well), and the luciferase activity was measured by using a Dual-Luciferase Assay System (Promega Corporation) and normalized to the internal control.

### Chromatin immunoprecipitation assays

**Figure 2:**
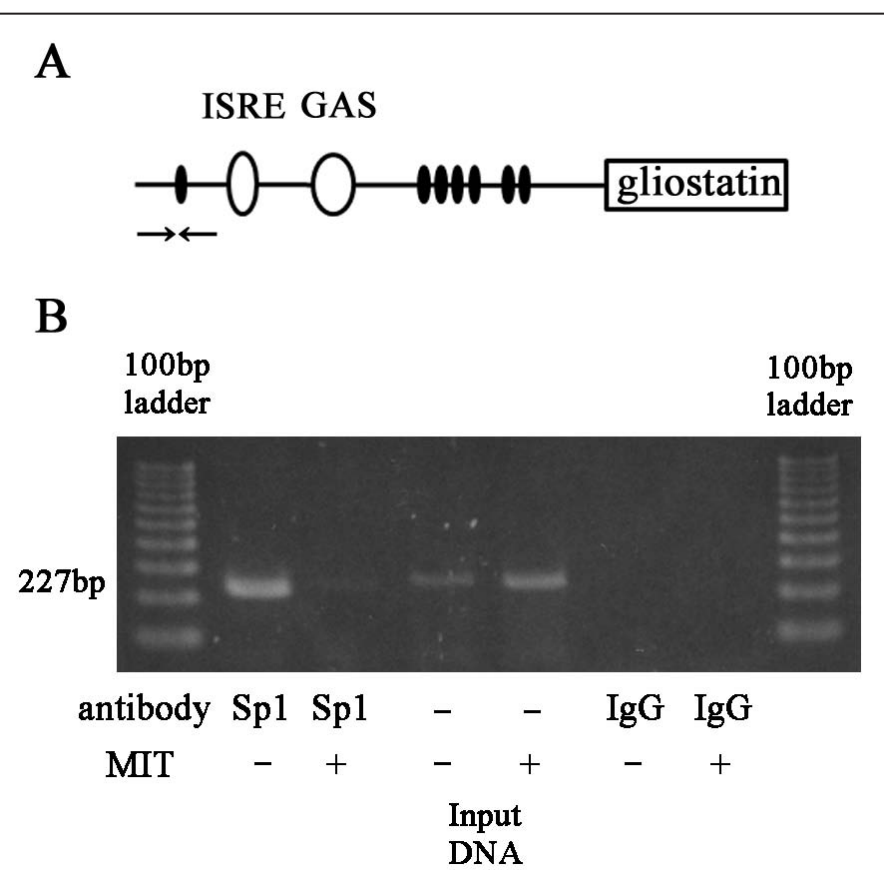
**Effect of mithramycin on Sp1 binding with chromatin immunoprecipitation (ChIP) assays**. Closed ovals indicate Sp1-binding sites. Open ovals indicate interferon-stimulated response element (ISRE) and gamma-activated sequence (GAS) sequences. Arrows indicate the polymerase chain reaction primers used for amplifying gliostatin/thymidine phosphorylase (GLS/TP) promoter containing the Sp1-binding site furthest upstream **(A)**. Confluent fibroblast-like synoviocytes were incubated in the presence or absence of 300 nM mithramycin for 24 hours. Sp1 binding to the furthest upstream Sp1-binding site within the GLS/TP promoter was detected by ChIP assays. This binding was suppressed by treatment with mithramycin **(B)**. Similar observations were obtained at least three times. bp, base pairs; MIT, mithramycin.

Chromatin immunoprecipitation (ChIP) assays were performed by using ChIP-IT™ Express kits (Active Motif, Carlsbad, CA, USA) in accordance with a reported protocol [27] with minor modifications. In brief, 1.0 × 10^6 ^FLSs cells from 60-mm dishes were treated with or without 300 nM mithramycin for 24 hours and then harvested. Cells were cross-linked by formaldehyde (1% final concentration) and incubated at room temperature for 10 minutes. Cells were then washed with ice-cold phosphate-buffered saline (PBS) containing protease inhibitors, and the fixation reaction was stopped by adding 10 mL of Glycine Stop-Fix Solution (Active Motif). Samples were lysed for 30 minutes in lysis buffer on ice, and the chromatin was sheared by sonicating 20 times for 40 seconds each at maximum power with an ultrasonic processor, including 30 seconds of cooling on ice between each pulse (Misonix, Inc., Farmingdale, NY, USA). Cross-linked and released chromatin fractions were immunoprecipitated with magnetic beads, Sp1 antibodies, and non-specific IgG (rabbit) on a rolling shaker overnight at 4°C. Cross-linking of the immunoprecipitates containing fragmented DNA was chemically reversed, and the polymerase chain reaction (PCR) was performed with MESA GREEN qPCR MasterMix Plus for SYBR Assay I Low ROX (Eurogentec, San Diego, CA, USA). The PCR primers used for amplifying promoters containing the Sp1-binding site furthest upstream were 5'-AACTGTGGGCCTTCCCACTC-3' and 5'-TGCTGAGGTCCTCGAAGAAAC-3', which produced a 227-bp fragment (Figure 2a).

### Western blot analysis for Sp1

FLSs were incubated in 60-mm dishes with 1 ng/mL TNF-α (R&D Systems, Minneapolis, MN, USA) in the presence or absence of mithramycin. To prepare nuclear extracts, cells were rinsed in ice-cold PBS and lysed in 10 mM Tris-HCl (pH 7.5), 1 mM ethylenediaminetetraacetic acid (EDTA), 0.5% Nonidet P-40, and a protease inhibitor cocktail (Sigma-Aldrich) for 10 minutes at 4°C. Cell lysates were centrifuged at 20,000 *g *for 10 minutes at 4°C to separate the cytoplasmic fraction (supernatant). Insoluble materials were dissolved in sodium dodecyl sulphate sample buffer to collect the nuclear extracts. After measuring the protein concentration, samples (20 μg of total protein/lane) were loaded and separated by electrophoresis on a 7.5% polyacrylamide gel (Bio-Rad Laboratories, Hercules, CA, USA) and then transferred to a polyvinylidene difluoride membrane (Immobilin-P; EMD Millipore, Billerica, MA, USA). The blots were blocked for 60 minutes with 5% non-fat dry milk in Tris-buffered saline containing 0.1% Tween 20 (TBS-T) and then incubated with an anti-Sp1 antibody (1:1,000; Cell Signaling Technology, Inc., Danvers, MA, USA), anti-lamin C (1:1,000; ImmuQuest Ltd, Seamer, North Yorkshire, UK), and anti-α-tubulin antibody (1:1,000; Cell Signaling Technology, Inc.) as loading controls in TBS-T for 2 hours at room temperature. After three washes with TBS-T, membranes were incubated with appropriate horseradish peroxidase-conjugated secondary antibodies in TBS-T (1:3,000; GE Healthcare, Little Chalfont, Buckinghamshire, UK) for 1 hour at room temperature and washed three times with TBS-T. Protein bands were detected by using the ECL Plus Western Blotting Detection System (GE Healthcare) and then quantified with a densitometric scanner by using ImageJ software [28].

### Reverse transcription-polymerase chain reaction assay

Expression of the GLS gene was assessed by using reverse transcription-PCR (RT-PCR). Total RNA was isolated by using TRIzol reagent (Invitrogen Corporation), and RT was carried out by using random primers and Ready-To-Go You-Prime First-Strand Beads (GE Healthcare). The resultant cDNA was subjected to real-time PCR by using a 7500 Fast Real-time PCR System (Life Technologies Corporation) with MESA GREEN qPCR MasterMix Plus for SYBR Assay I Low ROX (Eurogentec) and primers. The PCR conditions were an initial denaturation at 95°C for 10 minutes, followed by 40 cycles at 95°C for 5 seconds and 60°C for 1 minute. The relative quantification value of GLS was normalized to an endogenous control, β-actin, after confirming that the GLS and β-actin cDNAs were amplified with the same efficiency. The primers were as follows: GLS, 5'-ACAGGAGGCACCTTGGATAA-3' and 5'-CCGAACTTAACGTCCACCAC-3' (272 bp), and β-actin, 5'-GACCTGACTGACTACCTCAT-3' and 5'-TCGTCATACTCCTGCTTGCT-3' (542 bp).

### RNA interference

Sp1 Stealth siRNAs and negative control siRNA were purchased from Invitrogen Corporation. The following Sp1 siRNA oligos were used: sense sequence 5'-GACAGGUCAGUUGGCAGACUCUACA-3' and anti-sense sequence 5'-UGUAGAGUCUGCCAACUGACCUGUC-3'. FLSs were transfected with the indicated combinations of siRNA against Sp1 or negative control siRNA at a final concentration of 20 nM by using Lipofectamine RNAiMAX transfection reagent (Invitrogen Corporation) in accordance with the recommendations of the manufacturer. FLSs were harvested for Western blotting 48 hours after transfection. FLSs were incubated for 24 hours after transfection and further incubated with 1 ng/mL TNF-α for 24 hours and harvested for RT-PCR.

### Enzyme immunoassay for gliostatin

GLS was measured by using an enzyme immunoassay system, as described by Hirano and colleagues [29]. Polyclonal antibodies on the solid phase were obtained by immunizing New Zealand albino rabbits with 40 μg of purified natural human GLS, and the monoclonal antibody was used with the β-galactosidase-labeled secondary antibody. The detection limit of this assay was 150 pg/mL, and no significant cross-reactivity or interference was observed. The GLS concentration in cultured FLSs was normalized to the protein content, as measured by using a Pierce BCA protein assay kit (Thermo Fisher Scientific, Waltham, MA, USA).

### Immunocytochemistry

Confluent FLSs in chambered slides coated with BD Matrigel Matrix (BD Biosciences, Franklin Lakes, NJ, USA) were fixed for 30 minutes in 3% paraformaldehyde, permeabilized with 0.2% Triton X-100 for 5 minutes, washed with PBS, and blocked for 60 minutes at room temperature with blocking solution comprising 3% bovine serum albumin and 0.1% glycine in PBS. After washing, cells were labeled overnight at 4°C with the primary antibody (1:1,000 anti-TP/GLS antibody; a gift from Chugai Pharmaceutical Co., Ltd., Tokyo, Japan). Cells were washed and labeled with an Alexa Flour 594-labeled (red) goat anti-mouse IgG (1:1,000; Molecular Probes Inc., now part of Invitrogen Corporation) secondary antibody. After washing, sections were mounted on glass slides containing ProLong Gold Antifade with 4',6-diamidino-2-phenylindole (DAPI) (Invitrogen Corporation). Stained cells were visualized by using an AX70 fluorescence microscope (Olympus, Tokyo, Japan). Total red intensity of immunostaining in nine random fields was quantified by ImageJ. The numbers of cells were counted in the fields. Data were presented as mean of intensity per cell.

### Statistical analysis

All data were entered into an electronic database and analyzed by using GraphPad Prism 4 (GraphPad Software, Inc., San Diego, CA, USA). Data are expressed as the mean ± standard error of the mean unless otherwise stated. The statistical significance of the differences was examined by using the Mann-Whitney *U *test. In all cases, *P *values of less than 0.05 were considered to be statistically significant.

## Results

### Mithramycin inhibited gliostatin gene expression

We prepared three deletion constructs of the GLS/TP promoter and fused them to the reporter luciferase plasmid (Figure 1) to investigate whether mithramycin inhibited GLS/TP promoter activity. The promoter activity was expressed relative to the internal control. Treatment with mithramycin inhibited the promoter activity in RA FLSs transfected with pTP-Luc1 (55.5%, 100 nM mithramycin; 59.2%, 300 nM mithramycin), whereas promoter activity was measured at 41.8% in RA FLSs transfected with pGLS/TP I, relative to those transfected with pTP-Luc1. The observed inhibition was accelerated following treatment with mithramycin (38.2%, 100 nM mithramycin; 35.1%, 300 nM mithramycin) in RA FLSs transfected with pGLS/TP I. The promoter activity of pGLS/TP II and pGLS/TP III was largely inhibited with or without mithramycin.

### Analysis of the gliostatin/thymidine phosphorylase promoter by using a chromatin immunoprecipitation assay

We performed a ChIP assay to confirm Sp1 binding to the promoter and examined the inhibitory effect of mithramycin on GLS gene expression. Sp1 was shown to bind to the furthest upstream Sp1-binding site of the GLS/TP promoter fragment (1,243 bp). Treatment with mithramycin suppressed binding at this site (Figure 2b). No amplification band was observed by using the non-specific rabbit IgG, indicating the specificity of the DNA immunoprecipitation and ChIP assays.

### Nuclear accumulation of Sp1 in response to TNF-α and mithramycin

**Figure 3:**
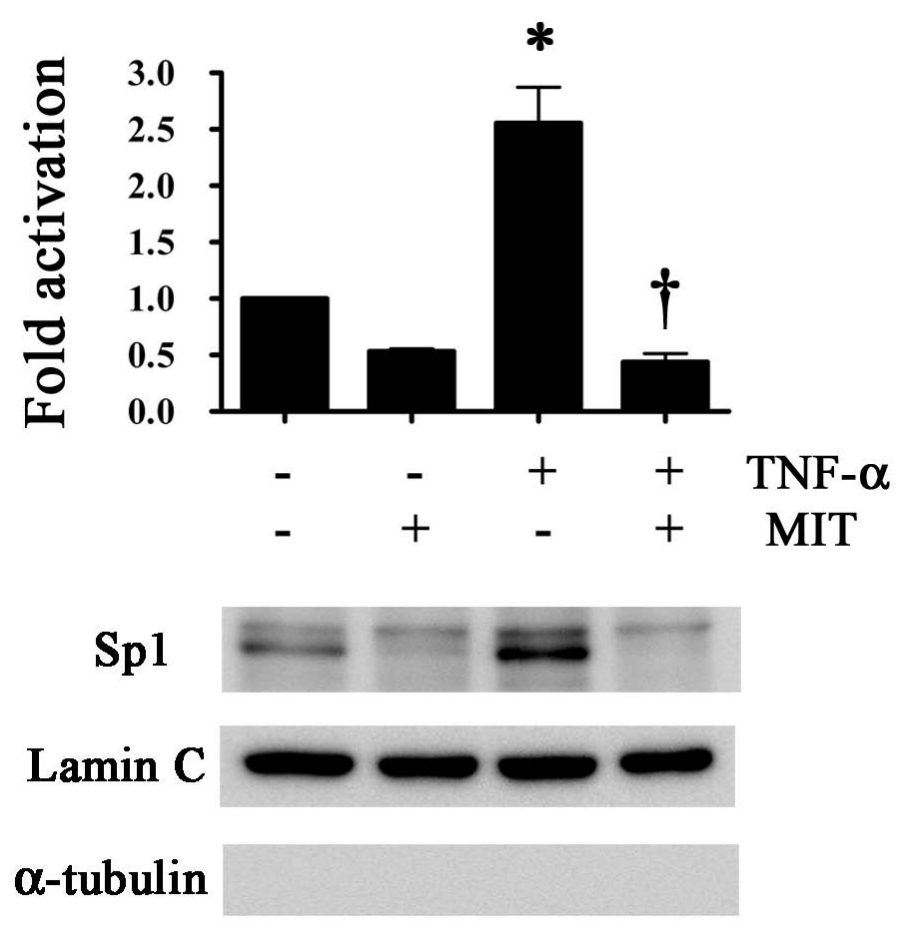
**Effect of tumor necrosis factor-alpha (TNF-α) and mithramycin on Sp1 protein expression in fibroblast-like synoviocyte nuclei**. Fibroblast-like synoviocytes were cultured to confluence in the presence or absence of 300 nM mithramycin for 30 minutes and then further incubated with or without TNF-α (1 ng/mL) for 24 hours. Nuclear extracts were processed for immunoblotting with an anti-Sp1 antibody. Anti-lamin C and anti-α-tubulin immunoblotting were included to assess the purities of nuclear and cytoplasmic fractions, respectively. Results are presented as mean ± standard error of the mean of five determinations. Statistical significance compared with controls was calculated by using the Mann-Whitney *U *test: compared with controls *P < 0.05; compared with samples with TNF-α alone ^†^*P *< 0.01. MIT, mithramycin.

To examine whether the nuclear localization of Sp1 changed following treatment with TNF-α or mithramycin, we prepared nuclear extracts from FLSs treated with or without 300 nM mithramycin for 30 minutes, further incubated with 1 ng/mL TNF-α for 24 hours, and then subjected to immunoblotting with anti-Sp1, anti-lamin C, and anti-α-tubulin antibodies. TNF-α treatment led to a remarkable accumulation of Sp1 in nuclear extracts, which was inhibited by treatment with mithramycin (Figure 3). The nuclear extracts were stained by the anti-lamin C antibody as a nuclear lamin marker but not by the anti-α-tubulin antibody as a cytoplasmic marker.

### Inhibition of gliostatin expression by RNA interference

**Figure 4:**
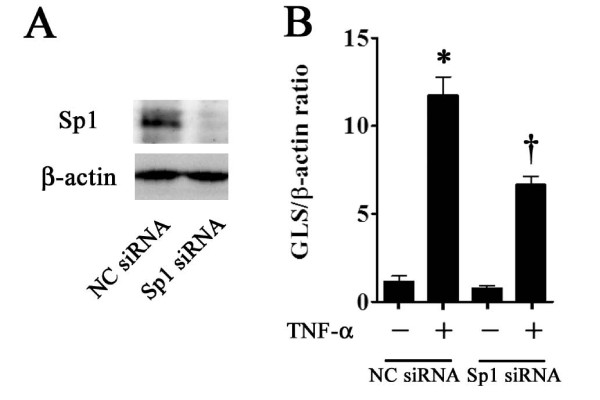
**Effect of Sp1 RNA interference on gliostatin (GLS) expression**. Western blotting analysis shows that Sp1 small interfering RNA (siRNA) (20 nM) transfection for 48 hours strikingly blocked Sp1 expression compared with negative control (NC) siRNA (20 nM) transfection **(A)**. After Sp1 or NC siRNA transfection for 24 hours, cells were treated with tumor necrosis factor-alpha (TNF-α) (1 ng/mL) for 24 hours. GLS mRNA expression levels are presented as mean ± standard error of the mean of five determinations **(B)**. GLS mRNA levels are represented relative to β-actin. Statistical significance was calculated by using the Mann-Whitney *U *test: compared with NC siRNA samples *P < 0.01; compared with NC siRNA samples with TNF-α ^†^*P *< 0.01.

To investigate the direct effect of Sp1 on GLS expression, we employed an siRNA against Sp1 for as an approach to inhibit GLS expression. The efficiency and specificity of siRNA gene knockdown of Sp1 were determined by Western blotting for Sp1 expression. Sp1 siRNA suppressed the Sp1 protein expression (Figure 4a). Sp1 siRNA reduced TNF-α-induced GLS gene expression by 56.7% at 48 hours post-transfection (Figure 4b).

### Effects of mithramycin on gliostatin production stimulated by TNF-α in cultured fibroblast-like synoviocytes

**Figure 5:**
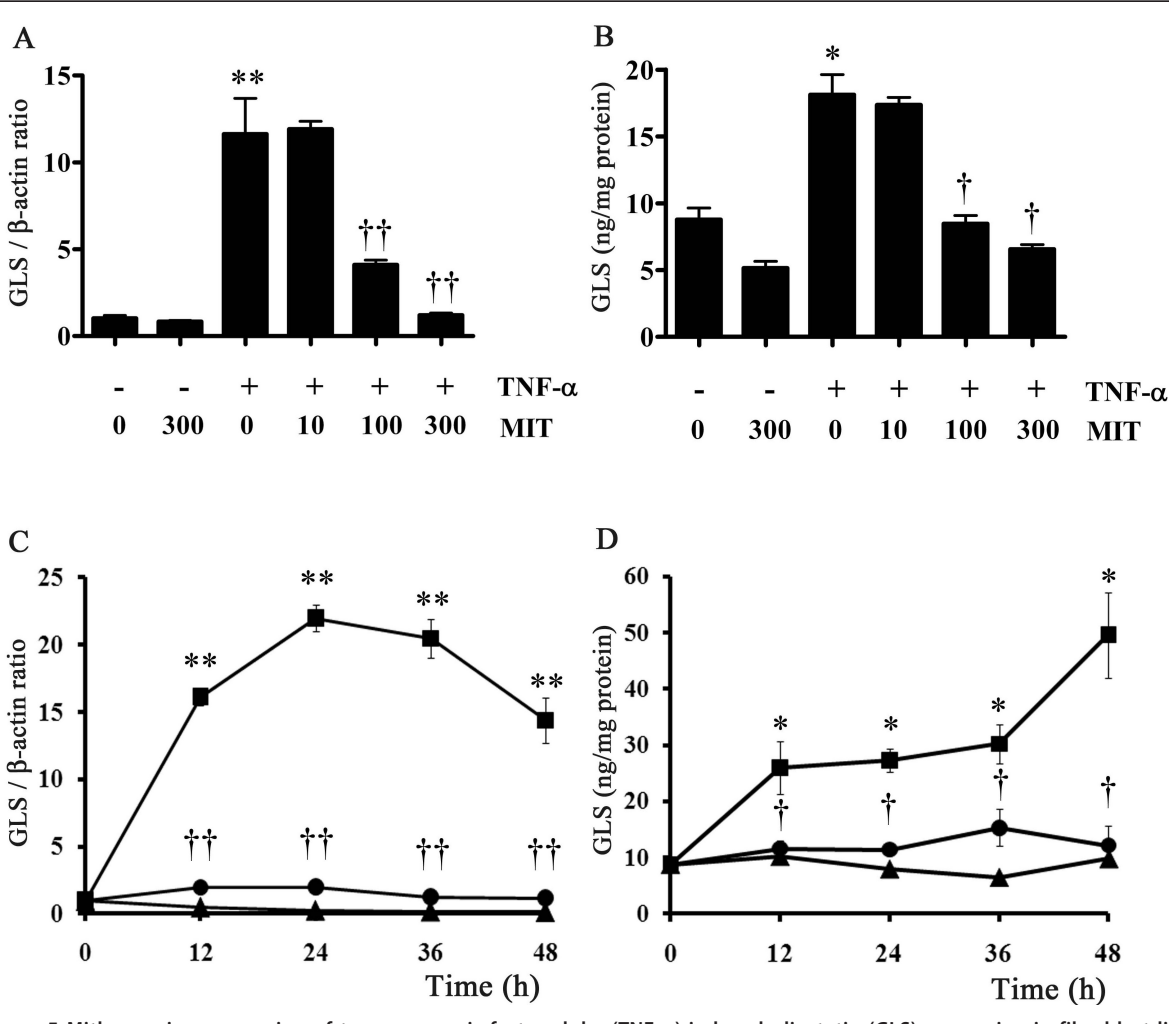
**Mithramycin suppression of tumor necrosis factor-alpha (TNF-α)-induced gliostatin (GLS) expression in fibroblast-like synoviocytes (FLSs)**. Confluent FLSs were incubated in the presence or absence of 10 to 300 nM mithramycin for 30 minutes and then with TNF-α for 24 hours. GLS gene expression levels are represented relative to β-actin **(A)**. Immunoreactive GLS was determined by enzyme immunoassay **(B)**. Results are presented as mean ± standard error of the mean (SEM) of four determinations. Confluent cells were incubated in the presence (closed circle) or absence (closed square) of 300 nM mithramycin for 30 minutes and then with TNF-α as indicated. Control FLSs were cultured without TNF-α (closed triangle). GLS mRNA **(C) **and protein **(D) **expression levels are presented as mean ± SEM of four determinations. Statistical significance was calculated by using the Mann-Whitney *U *test: compared with controls *P < 0.05, **P < 0.01; compared with samples with TNF-α alone ^†^*P *< 0.05, ^††^*P *< 0.01. MIT, mithramycin.

FLSs were treated in the presence or absence of 10 to 300 nM mithramycin for 30 minutes and then further incubated with 1 ng/mL TNF-α for 24 hours. GLS mRNA (Figure 5a) and protein (Figure 5b) levels were significantly induced by treatment with TNF-α alone (11.3-fold and 2.1-fold compared with the control, respectively), and these inductions were suppressed by mithramycin treatment in a dose-dependent manner. GLS was not induced by treatment with mithramycin alone. We confirmed the non-toxic concentration at least 48 hours after incubation with 10 to 300 nM mithramycin and 1 ng/mL TNF-α by using WST-8 assays (Cell Counting Kit-8; Dojindo Laboratories, Kumamoto, Japan) (data not shown). GLS mRNA expression increased in response to TNF-α (1 ng/mL) and reached a maximum at 24 hours (21.9-fold compared with the level at 0 hours) (Figure 5c). GLS protein expression increased in response to TNF-α and continued to rise until at least 48 hours after treatment (5.6-fold compared with the level at 0 hours) (Figure 5d). These increases in GLS mRNA and protein were significantly suppressed by 300 nM mithramycin treatment. To confirm whether TNF-α directly regulates GLS expression, FLSs were pre-treated with the protein synthesis inhibitor, cycloheximide. TNF-α-induced GLS mRNA production significantly decreased in a dose-dependent manner with treatment of cycloheximide (Additional file 1).

**Figure 6:**
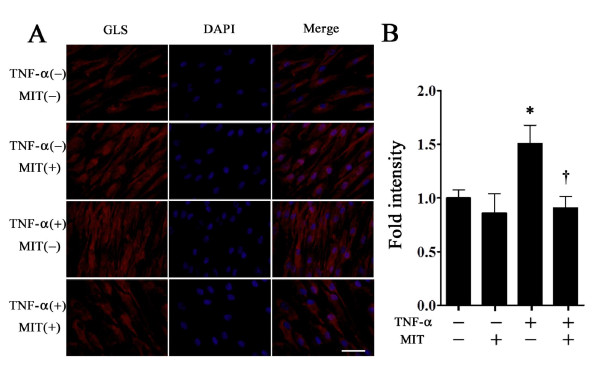
**Immunocytochemical detection of gliostatin (GLS) in fibroblast-like synoviocytes**. Fibroblast-like synoviocytes were stimulated by the presence or absence of 300 nM mithramycin for 30 minutes, followed by further incubation with or without tumor necrosis factor-alpha (TNF-α) (1 ng/mL) for 24 hours, and were immunostained with GLS antibody (red). Cell nuclei were stained by 4',6-diamidino-2-phenylindole (DAPI) (blue). Scale bar represents 50 μm **(A)**. Total red intensity of immunostaining in a random field was quantified by ImageJ. The numbers of cells were counted in the field. Data (intensity/cell) were presented as mean ± standard error of the mean (SEM) of nine determinations. Statistical significance was calculated by using the Mann-Whitney *U *test: compared with controls *P < 0.05, compared with samples with TNF-α alone ^†^*P *< 0.05 **(B)**. MIT, mithramycin.

FLSs were cultured to confluence in the presence or absence of 300 nM mithramycin for 30 minutes, followed by further incubation with or without 1 ng/mL TNF-α for 24 hours. We observed no morphological change in FLSs during immunocytochemical staining. FLSs were immunostained with GLS antibody (red). Staining was weakly diffuse in the cytoplasm of FLSs that had not undergone treatment (Figure 6a). Treatment with mithramycin alone had no effect on GLS staining. The expression of GLS protein was significantly induced by treatment with TNF-α alone (1.5-fold compared with the control). This induction was significantly suppressed by mithramycin treatment (0.6-fold compared with the sample induced by TNF-α alone) (Figure 6b).

## Discussion

TNF-α has been identified as a crucial cytokine in the pathogenesis of RA [30,31] and is involved in the activation of the nuclear factor-kappa-B (NF-κB) transcription factor [32,33]. In the present study, no NF-κB-binding sites were identified within the upstream GLS/TP promoter examined. It is therefore unlikely that TNF-α directly modulates transcriptional GLS regulation through binding the GLS/TP promoter. To determine the principal elements in the GLS/TP promoter which are relevant to GLS expression, we constructed a series of deletion mutant plasmids. Treatment with mithramycin inhibited GLS/TP promoter activity, confirming the potential for GLS production through Sp1 binding in FLSs.

Goto and colleagues [19] reported that interferon-gamma (IFN-γ) induced the expression of TP through ISRE and gamma-activated sequence in human macrophages. In the present study, the promoter activity was not regulated by IFN-γ in FLSs (data not shown); however, this discrepancy could be explained by differences in the cellular properties of macrophages and FLSs. In addition, luciferase and ChIP assays showed that the Sp1-binding sites played an important role in regulating the promoter activity of the GLS gene and mithramycin exactly behaved as an Sp1 inhibitor. Other reports suggested that activated Sp1 is transported into the nucleus [34,35]; this was supported by our analysis of FLS nuclear fractions by using Western blotting, which revealed a remarkable accumulation of Sp1 in the nuclei following TNF-α treatment that was inhibited by treatment with mithramycin. We further investigated whether Sp1 directly regulated expression of GLS. We used RNA interference to detect the effects of Sp1 depletion on GLS expression at the mRNA levels.

We confirmed that TNF-α-induced GLS mRNA and protein production were inhibited by mithramycin in a dose-dependent manner. Our immunocytochemical studies revealed that GLS was stained weakly and diffusely in FLS cytoplasm that had not undergone treatment. The intensity of GLS staining was increased by TNF-α treatment and suppressed by mithramycin at concentrations of 100 to 300 nM. These concentrations did not affect cell viability, as measured by a WST-8 assay, and an *in vivo *study demonstrated that leukemia patients receiving a 2-hour continuous infusion of 25 μg/kg mithramycin did not exceed plasma levels of 300 to 350 nM [36].

The protein synthesis inhibitor cycloheximide significantly decreased TNF-α-induced GLS mRNA production in a dose-dependent manner. GLS gene transcription might require *de novo *protein synthesis in FLSs stimulated by TNF-α, although the key protein involved remains to be identified.

In the pathogenesis of RA, we reported that GLS increased the expression of VEGF mRNA and protein in FLSs [14]. As angiogenesis is necessary for the perpetuation of inflammation, inhibition of angiogenic factors such as GLS and VEGF could provide a means to suppress the inflammatory cascade in RA synovitis [37]. We also reported that GLS induced the expression of matrix metalloproteinase (MMP)-1 and MMP-3 and GLS itself in FLSs [16]. The MMP family of proteins, including MMP-1, MMP-3, and MMP-13, plays a crucial role in excessive cartilage degradation in RA [38,39]. Other reports indicated that the gene expression levels of VEGF in fibroblasts [40,41] and of MMPs in articular chondrocytes [42] were closely related to that of Sp1. It was suggested, based on these findings, that an Sp1 inhibitor could be effective in reducing RA disease activity.

## Conclusions

The present study indicates that expression of the GLS gene is mediated, in part, through the transcription factor Sp1. Our data suggest that the beneficial effects of mithramycin in RA might be at least partly due to anti-angiogenic and anti-arthritogenic activity involving the downregulation of GLS. Mithramycin is therefore a promising candidate anti-rheumatic drug.

## Abbreviations

bp: base pair; ChIP: chromatin immunoprecipitation; FLS: fibroblast-like synoviocyte; GLS: gliostatin; IFN-γ: interferon-gamma; IL: interleukin; ISRE: interferon-stimulated response element; MMP: matrix metalloproteinase; NF-κB: nuclear factor-kappa-B; PBS: phosphate-buffered saline; PCR: polymerase chain reaction; RA: rheumatoid arthritis; RT: reverse transcription; RT-PCR: reverse transcription-polymerase chain reaction; siRNA: small interfering RNA; TBS-T: Tris-buffered saline containing 0.1% Tween 20; TNF-α: tumor necrosis factor-alpha; TP: thymidine phosphorylase; VEGF: vascular endothelial growth factor.

## Competing interests

The authors declare that they have no competing interests.

## Authors' contributions

KI and KK performed all the experiments, data analysis, and drafting of the manuscript. TY and MN participated in the interpretation of the data and performed the statistical analysis. MA and KA conceived of the study, participated in its design and coordination, and helped to draft the manuscript. YW-N conceived this study, provided financial support, designed experiments, interpreted the data, and drafted the manuscript. TO carried out administrative and financial support and helped to draft the manuscript. All authors read and approved the final manuscript.

## Supplementary Material

Additional file 1 **Effect of cycloheximide on TNF-α induced GLS mRNA production in FLSs**. Confluent FLSs in 6-well plate were incubated in the presence or absence of 10-100 (μg/ml cycloheximide for 30 min, followed by further incubation with TNF-α (1 ng/ml) for 24 h. The GLS mRNA levels are expressed as a RT-PCR product ratio (GLS/β-actin). Results are presented as mean ± SEM of four determinations. Statistical significance compared with controls was calculated using the Mann-Whitney U-test: compared to controls * *P *< 0.01; compared to samples with TNF-α alone † *P *< 0.01. Click here for file
