# Supplementary figures and images for: The Sp1 transcription factor is essential for the expression of gliostatin/thymidine phosphorylase in rheumatoid fibroblast-like synoviocytes
Source: Arthritis Res Ther. 2012 Apr 25;14(2):R87. doi: 10.1186/ar3811 (PMC3446461; doi:10.1186/ar3811)

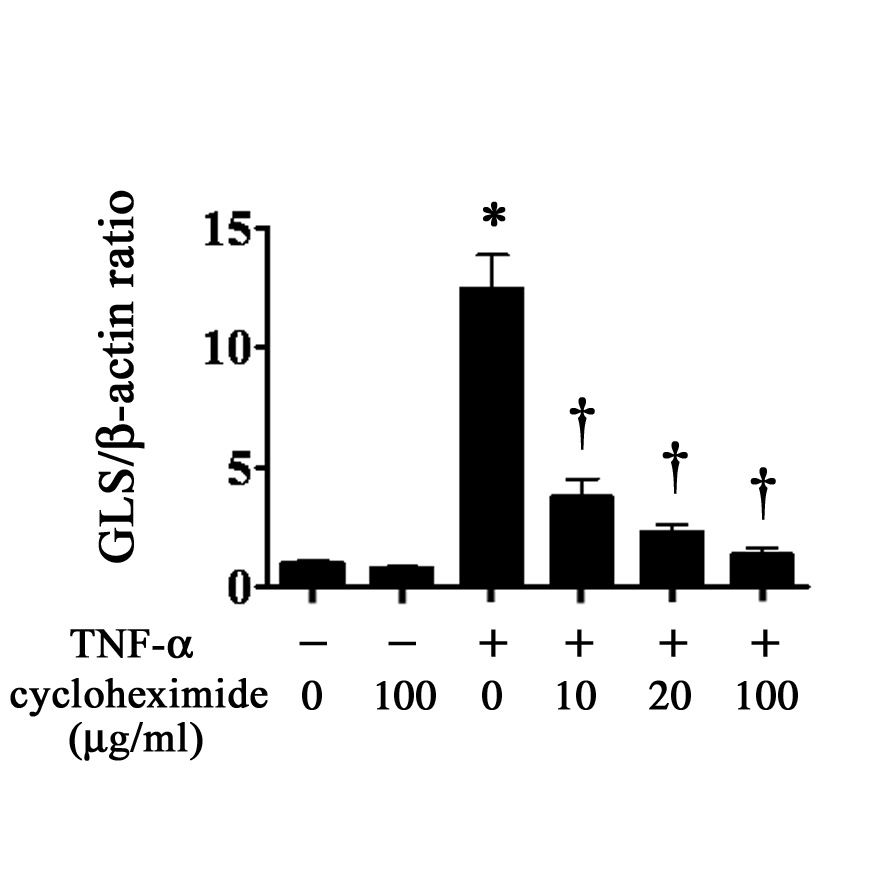

Supplement: Additional file 1 — Effect of cycloheximide on TNF-α induced GLS mRNA production in FLSs. Confluent FLSs in 6-well plate were incubated in the presence or absence of 10-100 (μg/ml cycloheximide for 30 min, followed by further incubation with TNF-α (1 ng/ml) for 24 h. The GLS mRNA levels are expressed as a RT-PCR product ratio (GLS/β-actin). Results are presented as mean ± SEM of four determinations. Statistical significance compared with controls was calculated using the Mann-Whitney U-test: compared to controls * P < 0.01; compared to samples with TNF-α alone † P < 0.01. [file ar3811-S1.TIFF]
